# Physical activity among children with down syndrome: maternal perception

**DOI:** 10.1080/17482631.2021.1932701

**Published:** 2021-06-02

**Authors:** Salmah Alghamdi, Maram Banakhar, Hanan Badr, Sanaa Alsulami

**Affiliations:** a Maternity and Childhood Nursing Department, Faculty of Nursing, King Abdulaziz University, Jeddah, Saudi Arabia; b Public Health Department, Faculty of Nursing, King Abdulaziz University, Jeddah, Saudi Arabia; c Faculty of Nursing, Umm AlQura University, Al-Qura, Saudi Arabia

**Keywords:** Down syndrome, physical activity, benefits, facilitators, barriers

## Abstract

**Purpose**: Studies have shown that children with Down syndrome (DS) are at high risk for physical inactivity and obesity. This study aimed to explore mother’s perceptions of the physical activity levels, needs, benefits, facilitators, and barriers in their children with DS.

**Methods**: For this descriptive qualitative study, 17 participants were recruited through centres for children with DS in the Kingdom of Saudi Arabia. Semi-structured interviews were conducted with mothers who were able speak Arabic or English and have a child with a confirmed diagnosis of Trisomy 21 (DS) between ages three and 17 years. Thematic analysis was used to analyse the study data.

**Results**: Data analysis revealed the following themes regarding children with DS: 1) their physical functioning level; 2) daily physical activity at home; 3) physical activity at school; 4) physical activity benefits; 5) physical activity facilitators; and 6) physical activities barriers.

**Conclusion**: Findings from this study can help health professionals gain insight on the physical activity facilitators and barriers for children with DS in order to design tailored intervention programmes to improve the support and the engagement of children with DS into regular physical activities.

## Introduction

Down Syndrome (DS) is one of the most common chromosomal disorders worldwide. In the USA, about 6000 newborns are born with down syndrome annually (Centers for Disease Control and Prevention, 2020). While in the UK, around 750 babies annually are born with down syndrome (Down’s Syndrome Association 2021). In the middle east, in particular, within Saudi Arabia, a study screened a total of 45,682 children and found that DS was the top one of the most common congenital anomalies with a prevalence rate of 6.6 per 10,000 (Salloum et al., 2015).

Children with DS have cognitive, speech, and communication delays as well as consistent patterns of physical inactivity that, over extended periods, result in reduced health-related quality of life (Alhusaini et al., 2017; Bendak, 2018; Ulrich et al., 2011). Childhood obesity is a major health concern among children with DS, increasing their moderate to vigorous-intensity physical activity (MVPA) participation could help prevent obesity and promote lifelong health in this population (Whitt-Glover et al., 2006).

The World Health Organization (2020) recommends that children should have a minimum of 60 minutes of (MVPA) daily. Unfortunately, meeting the recommended level of physical activity can be challenging for children with disabilities. Moreover, meeting the recommended daily MVPA becomes even more challenging for children with Down syndrome because they are at high risk for physical inactivity and obesity (Phillips & Holland, 2011). Therefore, strategies are needed to improve the quantity and quality of their physical activity.

The World Health Organization (2020) defines physical activity as any bodily movement generated by muscles and consumes energy. There has been a well-documented physical, cognitive (Biddle & Asare, 2011), psychological health benefits for physical activity. However, the majority of children with DS do not practice the MVPA recommended by their healthcare providers, and they are unlikely to participate in more than one sport (Barr & Shields, 2011; Lyons et al., 2015). According to a study conducted in the Kingdom of Saudi Arabia (KSA), compared to children without DS, children with DS exhibit sedentary behaviours and have less engagement in the recommended physical activity levels (Alhusaini et al., 2017). Furthermore, these authors found that children with DS aged eight to 12 years had a high body mass index (BMI) and higher levels of physical inactivity compared to the study’s control group (Alhusaini et al., 2017).

Previous researchers have explored physical activity facilitators and barriers for children with DS. Three commonly mentioned physical activity facilitators that encourage children with DS to participate more frequently in everyday activities were the positive role of the family, social interaction with peers, and available programmes that provide adaptations for children with DS (Barr & Shields, 2011; Lyons et al., 2015). In contrast, several barriers hindering children with DS from participating in physical activity across home, community, and social domains have been identified: the effect of common DS characteristics on maintaining an active lifestyle; competing family responsibilities; reduced physical and/or behavioural skills; and a lack of appropriate programmes serving children with DS (Barr & Shields, 2011; Lyons et al., 2015). A study performed by Shields et al. (2016) also affirmed that several DS characteristics limit children’s physical activity: foot posture, foot deformity, and poor footwear fit. In fact, Shields et al. (2016) concluded that poor footwear fit is associated with reduced physical activity in children with DS.

Parents play a major role in encouraging and supporting the physical activity of their children with DS (Pitchford et al., 2016). Furthermore, parents help in providing opportunities for their children with DS to develop motor and communication skills. Parents’ support, positive attitude, and knowledge all have been shown to affect the participation of children with DS in everyday activities (Lyons et al., 2015). According to Bricout (2015), from their children’s infancy, parents of children with DS need to be informed about positive-child rearing strategies that can minimize poor health status in their children. While children with DS experience health, motor, and communication impairments, they are able to learn how to engage in physical activities suitable to their health status.

Furthermore, parental perceptions of physical activity benefits are significantly associated with their children’s physical activity levels (Pitchford et al., 2016). While several studies have explored parental perceptions of physical activity for children with intellectual disabilities (McGarty & Melville, 2018; Pitchford et al., 2016), there is a lack of specific knowledge about maternal perceptions of the physical activity of their children with DS in the KSA. The perceptions of Saudi Arabian mothers are important to explore due to distinct sociocultural context.

Saudi Arabian mothers of children with intellectual disabilities experienced higher levels of stress compared to fathers, as they often work as the primary caregivers for their children (Aldosari & Pufpaff, 2014). Furthermore, there are a limited number of specialized centres for children with DS in the KSA (Alwhaibi & Aldugahishem, 2019). To the best of the authors’ knowledge, there is only one study by Alwhaibi and Aldugahishem (2019) that explored maternal perceptions’ regarding physical activity in children with DS in the KSA. However, it was limited to one city, Riyadh, and included only mothers of elementary school-aged children. Given the lack of research that examined maternal perceptions regarding physical activity in children with DS in the KSA, this study aimed to explore maternal perceptions of the physical activity levels, needs, benefits, facilitators, and barriers of their children with DS.

## Methods

### Design

This was a descriptive qualitative study. To collect comprehensive data, researchers conducted semi-structured, open-ended interviews with study participants.

### Ethical considerations

Institutional Review Board approval for the study was obtained from the Nursing Research Ethical Committee of the Faculty of Nursing at one university in the KSA. Internal approvals also were obtained from the participating centres before the mothers were contacted. Researchers ensured that all recruited mothers participated voluntarily, as they were given consent forms to sign before they were interviewed. Finally, participant confidentiality was maintained throughout the recruitment, data collection, data analysis, and findings reporting processes.

### Sample

A sample of mothers of children with DS across the KSA participated in this study. Mothers were included if their children had a confirmed Trisomy 21 diagnosis and were between three and 17 years of age. Children with chronic, acute medical conditions, psychological disorders, or physical disabilities that may hinder their physical activity were excluded.

Two sampling techniques were employed. First, convenience sampling was used to recruit mothers at four centres allocated in western region for children with DS after obtaining internal approval from centre staff. The centres distributed the study announcements and interested participants contacted the researchers. Next, a snowball sampling technique was used to recruit mothers of children with DS from different regions in the KSA, who were enrolled in either private or public schools. Participants also were recruited through a flyer posted on social media (Facebook and WhatsApp), after which interested mothers were approached by the researcher.

Considering the study sample size, from the 30 mothers who were initially contacted, a total of 9 individual interviews were conducted. Nevertheless, due to the insufficient generation of data, an additional 8 interviews were conducted. Therefore, the final sample size included was 17 participants. Moreover, the remaining participants (mothers) were not interviewed as the researchers reached data saturation when no additional information or themes emerged from data analysis.

### Data collection

Semi-structured, face-to-face interviews were conducted. However, because the KSA covers a wide geographical area, conducting some interviews over the phone made it possible to have broader participation. Participants were informed before starting the interviews that their participation was voluntary and they could withdraw at any time without penalty. The researcher also obtained the participants’ approval to record the interviews.

To collect comprehensive data, a semi-structured interview guide was developed based on the literature including about seven predetermined questions and allowing the interviewers to dig deeper and ask probe questions. Interviews were begun with an opening prompt “tell me about your child, including their age, sex, and level of physical functioning.” followed by further prompts. The mothers were interviewed individually, and the interviews were recorded. The interviews lasted 30 to 60 minutes.

### Data analysis

The data collection and analyses were conducted simultaneously as the recordings were transcribed verbatim with notes after each interview and analysed by using the six steps of thematic analysis (Polit & Beck, 2018). In the first step, researchers reviewed all the transcripts and then sent them to the participants for checking in order to ensure the accuracy of the transcribed interviews. Furthermore, to maintain anonymity and confidentiality of the data, the codes were given for each included participant. In the next step, all the research team started to familiarize themselves with the data by reading the interview transcripts line by line searching for the initial codes. In step 3, a coding table was created which involved initial codes, potential themes and sub-themes. In step 4, after the authors’ discussions regarding the interview data, all researchers returned to the interview transcripts and continued to identify themes and sub-themes. In step 5, the analysed interviews were verified and discussed by all researchers and the final themes and subthemes were identified. As a last step, the participants’ quotations were reviewed and selected to elucidate the identified themes and sub-themes. Examples of codes, subthemes and themes are presented in Table I.

**Table I.** Overview of examples of codes, subthemes and themes

Example of initial code	Example of subtheme	Example of final theme
Very active Always active Obese Sitting with the Technological devices Bored Tired	Positive and Negative Maternal Perspectives on Physical Activity of Children with DS	Physical Functioning Level
Climbing Playing football Dancing Biking Playing Swimming	Home Physical Activity	Daily Physical Activities at Home
Strengthen the muscles Prevent obesity Explore new things and places Feeling happy Improve mental health	Physical and psychological Benefits	Physical Activity Benefits
Encouragement Social acceptance Social interaction Awareness	Family Encouragement Opportunity for Social Interactions	Physical Activity Facilitators
Difficult transportation Low income Health problems Lack of support Lack of opportunities Age	Family Income Medical issues Supportive services Enrolment criteria	Physical Activity Barriers

The validity and trustworthiness of the study’s qualitative findings were assured through several methods. The researchers asked all participants to provide member checking by either confirming or clarifying the information they provided during the interviews. Furthermore, the mothers corroborated that the themes derived from the data represented an accurate description of their beliefs and perceptions. All authors participated in this study had previous experiences in conducting qualitative research; thus, they have the skills to explore the language, clarify the questions, and employ the aspects of active listening during the interviews. All authors were involved simultaneously in the process of thematic analysis and interpretation to confirm the interpretations from different perspectives.

## Results

**Figure 1.**
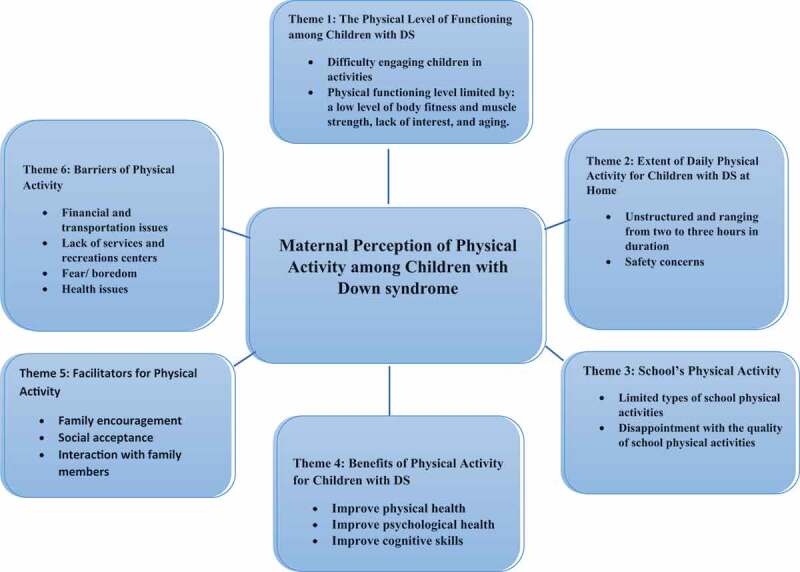
Summary of maternal perception of physical activity among children with Down syndrome

The final sample consisted of 17 mothers of seven male and ten female children with DS who lived in different regions across the KSA. All 17 mothers are Saudis and married. The majority had at least a high school certificate. The mothers also represented different age-groups ranged in age from 26 to 53 years old and their children ranged in age from three to 18 years old. Out of 17, 14 mothers are not working, and 7 mothers received a monthly income ranging from 4000–8000 Saudi riyals (Table II). Data from the 17 interviews produced six salient themes described below (Figure 1).

**Table II.** Participant socio-demographic characteristics (N = 17)

Characteristic	Frequency N (%)
Mother’s age20–2930–3940–4950–60	1 (5.8)2 (11.7)10 (58.8)4 (23.5)
Maternal employment statusWorking full-timeWorking part-timeStudentNot Working	2 (11.76)1 (5.88)0 (0.00)14 (82.35)
Number of People living in household2–45–78–10More than 10	1 (5.8)11(64.7)4 (23.5)1 (5.8)
Mother’s monthly income3000 or less SR4000–8000 SR8001–13,000 SRMore than 13,000Don’t know	1(5.8)7(41.2)7(41.2)1(5.8)1(5.8)
Maternal educational levelBelow high schoolHigh schoolSome collegeBachelor’s degreeMaster degree or higher	1(5.8)7(41.2)1(5.8)8(47.1)0
Child’s age3–67–1011–1415–17	(35.3) 6(41.2) 7(17.6) 31 (5.8)
Child’s genderMaleFemale	7 (41.2)10 (58.8)

### Theme 1: physical functioning level

While most participants expressed positive perceptions of their children’s physical activity, others expressed negative perceptions of it. The majority of mothers saw their children as naturally active and agreed on the importance of physical activity and its health benefits for their children with DS. One mother said, “he has been very active since he was 11 years old.” Another mother noted, “my daughter is always active, we cannot catch her.” On the other hand, a few mothers believed that their children’s loss of functional ability was linked to their lack of body fitness and muscle strength, which can contribute to obesity. One mother emphasized this issue by saying, “I am very worried about my child of getting obese; children with DS are always hungry.”

Although all mothers wanted their children to participate in physical activity to maintain their functional abilities and independence, some had difficulty engaging their children in such activity. These mothers felt their children spent more time using technology, which took away from time spent in physical pursuits. For example, one mother said that “kids now are sitting with technological devices most of the time. For instance, they are only eating and sitting in front of the TV and they then will gain weight.”

Another difficulty mentioned by mothers was that their children get bored easily and lose interest in activities. One mother said, “my son is easily feeling tired and quickly got bored with staying indoors.” Two other mothers also noted that because their daughters get bored from time to time, they are not getting enough exercise.

#### Subtheme 1: the association between physical activity and age

The mothers of preschool children felt it was easy to keep their children active, as they were always playing. However, mothers of children aged seven to 10 years often described their children as “stubborn” and “obstinate.” These mothers noticed that, during their middle childhood years, their children did not want to go outside, play, or go to bed. For example, one mother stated, “she is not listening, we need to ask her several times to do the activities. She is very stubborn.”

### Theme 2: daily physical activities at home

When participants were asked how much and what types of physical activity their children participated in every day, their answers were grouped into the following two categories: the home-based physical activity programmes used and the duration of the physical activities the children performed daily.

All the activities the mothers provided for their children were considered unstructured indoor activities. Participants estimated that their children were physically active an average of two to three hours per day after school hours. The most commonly reported everyday activities were playing football, walking outdoors, dancing, biking, swimming, and following workout videos. A few mothers described how their children helped them clean up the house and prepare some easy meals. One mother said of her son, “he is always helping me in picking up all the things he found on the floor and returning them back to the right place.” Finally, several mothers expressed their worries regarding some dangerous activities their children performed at home, such as climbing in closets or cabinets and playing in the toilet. One mother said, “my son is very kinetic, he is also climbing the bedroom closets.”

### Theme 3: physical activity at school

For Theme 3, participants described the type and appropriateness of physical activities provided by the schools for their children.

#### Subtheme 1: types of school activities

The majority of participants reported that their children participated in the following school activities: routine morning exercises, sewing, cooking, walking outdoors, and going to the outdoor play area to use the swings and slides. These mothers reported that their children spent an average of two hours in physical activity during the school day.

Some participants had negative experiences regarding the availability of various physical activities at schools. For example, one mother stated, “during the school day, a few morning exercises are the only thing provided for our children.” Another mother reported swimming as an example of a school activity. All mothers reported having no impact or input on the amount and type of physical activity at the school.

#### Subtheme 2: appropriateness of school activities

Because children with DS have a range of physical difficulties that can impact their motor development, many participants discussed the suitability of the physical activities provided by the schools. In particular, several participants noted that not every activity that is successful for most children would be appropriate for children with DS. In physical education practices, the mothers believed that children with DS always require close attention, careful instruction, and quality assurance in their activities.

The mothers also recognized the importance of making physical activities a part of their children’s everyday life. However, they noted that only the children aged seven years and older were getting involved in the school’s physical activities. For example, one mother said, “the school team does not involve the preschool-aged children with DS in the activities. Once they become 10 years old, they get involved.”

### Theme 4: physical activity benefits

All study participants emphasized the physical and psychological benefits of physical activity for children with DS. All participants agreed that physical activity can improve their children’s health by strengthening their muscles and stimulating blood circulation. One participant said that physical activity is good for “muscle strengthening, straightening, tightening, and it is good for blood circulation.” The majority of participants’ main concern was to involve their children in physical activity in order “to prevent obesity and help them maintain physical fitness.” Of note, one participant stated that physical activity helped enhance her child’s cognitive skills, “physical activity is very important, because it allows him to explore other new things, even at home he loves to explore places and grow something in his head, and develop something in his thinking.”

Regarding psychological benefits, participants noted that physical activity and exercise can enhance the mental health of children with DS, prevent depression, and stimulate feelings of happiness. One participant noted, “physical activity helps to improve the child’s mental health and prevent depression.” Additionally, participants reported that such activity had a significant impact on the children’s mood, energy, and behaviour. One participant said, “it helps to release the energy, modify the behaviour, and develop their intelligence.”

### Theme 5: physical activity facilitators

Participants reported on three key physical activity facilitators for their children: family encouragement, social acceptance, and social interaction with family members. The data indicated that family awareness plays a key role in motivating and encouraging children with DS to participate in physical activity. One mother noted that “family encouragement and awareness is very important in dealing with children with DS.” Furthermore, another participant emphasized the importance of “being patient and being repetitive to encourage children with DS to engage in physical activity.”

The majority of participants agreed that being socially understood and accepted in the community also is important for their children. For example, one participant said, “the first thing is that people outside the home must understand his condition … [then], even if his activity increases or his righteousness increases, no one will feel sorry for him.” Another mother emphasized that giving her child the opportunity to interact and play with family members serves as a facilitator for physical activity. She said, “playing with her siblings and other family members makes her feel happy.”

### Theme 6: physical activity barriers

Participants discussed different barriers that prevent their children with DS from being physically active. A family’s financial status (e.g., “low family income”) could affect their ability to register their children at DS centres and transport them there. Two participant comments echoed by several mothers were “financial status is important, as I can’t pay” and “I have a transportation problem.” Moreover, not all the participants were aware that a child having DS could affect a family’s ability to care for and deal with that child. One of the participants stated that the awareness campaigns conducted by DS centres or medical professionals are very important for increasing DS awareness and knowledge. She said, “Awareness campaigns performed by medical students on Down syndrome helped me to understand the condition and how to care for my child.”

All the mothers cited their children’s health and medical issues as the most significant barrier to them engaging in physical activity. Several participants noted that health problems (e.g., obesity, osteoporosis, and heart problems) prevent children with DS from exercising. One mother described her son’s problems; he has “weakness of his muscles, health problems, such as heart, leg, and knee problems and osteoporosis.” Another stated, “Sometimes obesity may be the reason that the movement is heavy.”

Many mothers agreed that there is a lack of appropriate supportive services for the children with DS. Participants stressed their need for an intervention programme led by professionals (e.g., physiotherapists, nutritionists, and speech therapists). For example, some participants reported that the centres and schools their children with DS attended did not employ specialists who know how to work with children with DS and accommodate their physical needs.

Another commonly cited barrier was boredom. It seems that children with DS become bored easily when exercising, dancing, or even playing with toys. One mother said her daughter is often “feeling bored when you play a game with her, she plays once alone, and she gets angry and screams when I tell her to build blocks.”

Another barrier was participants’ fear of their children with DS being around children without disabilities. Specifically, the mothers noted that the way their children with DS played and engaged in physical activity was affected by how other children felt about them and whether or not they accepted them. One participant reported having a “fear of crowding, lack of acceptance from the others, and people … looking at my child like he is an abnormal and a strange child.” Another mother added, “Children gathered against him and started to resist, and he thought that they were playing with him, and they started to be afraid of him.” Participants also reported on two other barriers: the absence of specialized centres for children with DS and the lack of acceptance their children experienced in the available centres. One mother stated, there are “no adequate centres and programmes for children with DS, as well as lack of opportunity for physical activity.” Furthermore, participants reported that there are few fitness centres or exercise programmes that serve the children with DS.

All mothers also agreed there is a lack of recreational activities that children with DS can perform as leisure-time physical activities. Because families understand the health benefits associated with regular physical activity, typically they are supportive of physical activity for their children with DS. While many of the participants had tried to register their children at available community and fitness centres, those centres often refuse to accept children with DS. This rejection negatively affected the type and level of physical activities performed by children with DS. Furthermore, all the mothers described another barrier: the lack of gym centres for people with special needs that offer structured physical activity programmes led by professionals experienced working with the children with DS. Of this barrier, one participant said, “my son likes to walk, run, and play soccer games. I wish he could be able to swim; he likes swimming and is very interested to learn how to swim. However, where are the fitness centres that can accept children with DS?

## Discussion

This was a descriptive qualitative study aimed to explore maternal perceptions of the physical activity levels, needs, benefits, facilitators, and barriers of children with DS. The study’s sample was representative, including mothers of children with DS enrolled in private or public centres. In the current study, the participants were from different regions within the KSA. The diversity of the ages and settings represented different points of views. This study revealed important findings about the physical activity of children with DS from Saudi Arabian mothers’ perception.

Participants had positive perceptions of their children’s physical activity, and described them as physically active, that is congruent with perceptions of parents in a Western study (Menear, 2007). However, the participants reported that their children’s physical functioning level could be limited by three factors: a low level of body fitness and muscle strength, the lack of interest, and being at school-age (older than seven years). Aligned with this finding, previous qualitative studies consistently have cited muscle weakness in children with DS as a factor limiting their physical activity (Alesi & Pepi, 2017; Alwhaibi & Aldugahishem, 2019). Developing programmes to strengthen the muscles of children with DS is necessary for enhancing their physical activity levels.

Mothers described their children’s daily physical activity as unstructured and ranging from two to three hours in duration such as playing football, walking outdoors, and dancing However, this amount and intensity of physical activity performed by the children with DS ranged from light to moderate, thus they are not achieving the recommended amount and intensity of physical activity described by the World Health Organization (2020) as 60-minutes of moderate to vigorous-intensity physical activity.

The mothers also had serious concerns about their children performing unsafe actions (climbing furniture). These concerns about safety during physical activity, which can be related to their children’s cognitive limitations, aligned with other studies indicating similar parental concerns as a barrier for physical activity (Alwhaibi & Aldugahishem, 2019; Barr & Shields, 2011; McGarty & Melville, 2018). While there might be a need for parents to directly observe their children during physical activity, such a practice may limit the type and duration of home-based physical activities. Consequently, efforts should be taken by nurses and specialists to support home-based physical activities through ensuring the application of home safety measures.

Participants noted that the ageing of children with DS limits their physical activity. This finding is congruent with that of other studies showing that children with DS became less active as they get older (Barr & Shields, 2011; Downs et al., 2013; McGarty & Melville, 2018). This decrease in the level of physical activity with ageing may be due to increased body weight and musculoskeletal problems that are common among children with DS as they get older (Downs et al., 2013; Esbensen, 2010). Although physical activities are important for children of all ages, mothers in the current study reported that their children had limited engagement in school physical activities. Therefore, regular engagement and monitoring for children with DS as they get older is crucial to maintain their fitness and muscle strength.

Findings from this study indicated that the types of school physical activities are limited. The majority of mothers were disappointed regarding the quality of school physical activities for their children, and the fact that they had no influence to change the school activity plans or types. Consistently, findings from a similar study in the KSA revealed the frustration of mothers due to difficulty of finding facilities that support the physical activity of children with DS (Alwhaibi & Aldugahishem, 2019). Another study conducted in the USA revealed similar issues, that the parents had no influence on the type and the amount of school physical activities (Menear, 2007). There seems to be a disconnection between children’s needs, as identified by their parents, and the scheduled school activities. Thus, a tailored, school-based physical activity programme should be developed for children with DS that considers their needs and preferences.

Participants discussed the perceived physical benefits of physical activity, including muscle strength, blood circulation, and obesity prevention. The psychological benefits that mothers reported were depression prevention and mood improvement. The mothers’ understanding and perception of the benefits of physical activity for children with DS is important because these children have a high risk of developing obesity (Menear, 2007; Whitt-Glover et al., 2006), which negatively affects their overall health. Lyons and colleagues (2015) supported the same findings because their study focused on parents’ perceptions of the benefits, facilitators, and barriers affecting the participation of children with DS in daily physical activities. It was found that participation in physical activities improved the children’s skills, promoted their psychological well-being, and boosted their sense of belonging in the community (Lyons et al., 2015). In addition, their study found that physical activity improved the children’s communication skills and empowered them to participate in their community and environment (Lyons et al., 2015).

Three categories of facilitators were identified through data analysis, all of them centred on the social factors, such as family and social acceptance. These categories were consistent with the findings from other studies. Two of the physical activity facilitators identified by Barr and Shields (2011) were similar to our findings: the positive role of the family and social interaction with peers. The positive family role dealt with siblings being positive role models to encourage physical activity (Barr & Shields, 2011). Likewise, Pitetti et al. (2013) also considered positive social and family attitudes as environmental factors that facilitate the engagement of children with DS in physical activity.

The results of the current study were consistent with the findings of Alwhaibi and Aldugahishem (2019) that was conducted in the KSA. Similar four themes emerged concerning the facilitation of physical activity for children with DS, these were mothers’ support and sibling involvement, peer involvement, and experience. Alwhaibi and Aldugahishem (2019) found that children with DS like to imitate their mothers and siblings in their physical activity, which helps keep the children more active.

The current study’s participants reported facing several barriers related to their children’s participating in physical activity: the children’s health and medical issues, the family’s financial status, lack of transportation, lack of services, lack of recreational activities, and fear of engaging in activities with others. Pitetti et al. (2013) divided the barriers into personal and environmental barriers, with the personal barriers (health problems, low fitness levels, and low motor skills) being similar to current study findings regarding health and medical issues.

Barr and Shields (2011) reported similar findings regarding the barriers of DS characteristics and common health problems with weight, physique, cardiac function, communication, and cognition. These findings were reflected in the current study as children with DS feel bored while engaged in physical activities. Alwhaibi and Aldugahishem (2019) reported similar findings discussing the most common health conditions associated with Down syndrome, specifically musculoskeletal, cardiovascular, and respiratory health problems that are affected by the level of the physical activity. Cardiovascular disease affects between 44% and 58%, and respiratory disorders affect about 36% of children with DS worldwide (Weijerman & De Winter, 2011). Limited physical activity between the ages of five and seven is common among these children (Weijerman & De Winter, 2011). In the current study, some of the mothers mentioned that their children’s walking was delayed until the age of three to five years, which was consistent with the findings of the other studies (Menear, 2007; Weijerman & De Winter, 2011).

Negative attitudes and stereotypes also were mentioned in the current study findings, expressed as children with DS experiencing a lack of acceptance from other children, their families, and some recreation centres, which refused to enrol children with DS, or required high fees for this population. Consistently, other studies reported the same results and categorized these results under social barriers (Alwhaibi & Aldugahishem, 2019; Barr & Shields, 2011). Lyons et al. (2015) mentioned that while a summer camp employee had a positive attitude towards children with DS, some extended family and community members had negative attitudes towards the abilities of children with DS (not wanting to take care of them). A possible explanation for these findings is the lack of awareness and knowledge in dealing with children with DS. At the summer camp, while the employees should be trained in working with children with DS, lay people still might lack general DS information, and the skills needed to work with this population.

Financial status and the lack of transportation have been found to be barriers. Barr and Shields (2011) mentioned competing family responsibilities, which included costs and finances. Alwhaibi and Aldugahishem (2019) also found that access to transportation was a barrier to physical activity for children with DS. Although Alwhaibi and Aldugahishem (2019) did not specifically mention financial difficulties, they did note that the unavailability of cheap public transportation and the cost of a private driver as barriers that prevented some of the families from enrolling their children in recreation centres. In the current study, it was found that admission fees to private centres and health service costs are the primary financial burdens for families of children with DS. Suggested solutions to overcome the financial burdens include providing financial support, and decreasing admission fees at specialized centres, particularly for low-income families.

The lack of accessible programmes and recreation centres has been also identified as barriers to physical activity for children with DS in other studies (Alwhaibi & Aldugahishem, 2019; Barr & Shields, 2011). Alwhaibi and Aldugahishem (2019) mentioned that in Saudi Arabia these facilities are available only in the major cities like Riyadh, Jeddah, and Dammam. In our study one of the participating mothers mentioned that the family moved from Makkah to Jeddah to be close to the DS centre.

Although our study identified six themes, some themes that were found in other studies did not emerge in the current study. Barr and Shields (2011) found competing family responsibilities, time constraints, supervision requirements, and the lack of parental involvement to be factors affecting the physical activity of children with DS. Furthermore, Alwhaibi and Aldugahishem (2019) mentioned that the Saudi culture requires the mother to maintain regular visits to her family and her husband’s family, which might occur on a weekly basis. Such visits take a lot of time and prevent the family from engaging in other activities. This issue might be a problem for some families; however, the presence of social support from different family members, and the presence of housemaids help the mothers manage their time and care for their DS children.

### Limitations

Although the participants were recruited from different centres across the KSA, the transferability of this study’s results may be limited as a result of including a small number of participants and by the fact that the participants were only mothers, with fathers not included.

Another limitation is that the majority of mothers participating in the study were recruited from private centres.

### Implications and future research

The results of our study emphasized the importance of increasing awareness about the need of children with DS for physical activity, particularly targeted programmes supported by government and private sectors. For future studies, it is suggested to replicate this study in different regions of the KSA, to allow a comparison of findings to determine whether barriers and facilitators differ based on region. Future research also should include both parents in order to explore variations in the viewpoints among mothers and fathers. Moreover, including both parents could indicate whether educational programmes should involve both parents, or if one parent is sufficient in caring for a child with DS.

## Conclusion

This study resulted in the identification of six themes regarding physical activity in children with DS: their level of physical functioning, daily physical activity at home, physical activity at school as well as physical activity benefits, facilitators, and barriers. All of these findings were consistent with those of other studies conducted in other countries. Furthermore, these findings indicate that children with DS and their mothers are suffering from similar concerns and barriers that are not being resolved by the stakeholders responsible for providing adequate services for these children. Therefore, these findings also serve to highlight needed action to address the problems described here.
